# Elevated TAK1 augments tumor growth and metastatic capacities of ovarian cancer cells through activation of NF-κB signaling

**DOI:** 10.18632/oncotarget.2273

**Published:** 2014-07-27

**Authors:** Patty C.H. Cai, Lei Shi, Vincent W.S. Liu, Hermit W.M. Tang, Iris J. Liu, Thomas H.Y. Leung, Karen K.L. Chan, Judy W.P. Yam, Kwok-Ming Yao, Hextan Y.S. Ngan, David W. Chan

**Affiliations:** ^1^ Department of Obstetrics and Gynaecology, LKS Faculty of Medicine, The University of Hong Kong, Hong Kong SAR, P.R.China; ^2^ Department of Biochemistry, LKS Faculty of Medicine, The University of Hong Kong, Hong Kong SAR, P.R.China; ^3^ Department of Pathology, LKS Faculty of Medicine, The University of Hong Kong, Hong Kong SAR, P.R.China

**Keywords:** TAK1, NF-κB signaling, ovarian cancer, high-grade tumor

## Abstract

Transforming growth factor (TGF)-β-activating kinase 1 (TAK1) is a serine/threonine kinase which is frequently associated with human cancer progression. However, its functional role in tumorigenesis is still controversial. Here, we report that TAK1 enhances the oncogenic capacity of ovarian cancer cells through the activation of NF-κB signaling. We found that TAK1 is frequently upregulated and significantly associated with high-grade and metastatic ovarian cancers. Mechanistic studies showed that Ser412 phosphorylation is required for TAK1 in activating NF-κB signaling and promotes aggressiveness of ovarian cancer cells. Conversely, suppression of TAK1 activity by point mutation at Ser412, RNAi mediated gene knockdown or TAK1 specific inhibitor ((5Z) -7-Oxozeaenol) remarkably impairs tumor growth and metastasis in ovarian cancer *in vitro* and *in vivo*. Our study underscores the importance of targeting TAK1 as a promising therapeutic approach to counteract the ovarian cancer progression.

## INTRODUCTION

Ovarian cancer is one of the leading causes of cancer death in females worldwide [1]. The high mortality rate of this disease is due to its late diagnosis and therefore poor prognosis as most cases present with aggressive ovarian cancer [2]. Despite advances in cancer treatment in the past decade, the cure rate of this disease remains modest [3]. Recent studies have revealed that personalized gene-targeted cancer therapy shows promising results in improving the survival and quality of life of patients suffering from aggressive tumors [4-7]. Therefore, a better understanding of the molecular mechanisms underlying ovarian cancer oncogenesis is urgently needed for guiding this therapeutic approach.

It is well-known that nuclear factor-kappa B (NF-κB) signaling pathway has multiple roles in cancer progression such as anti-apoptosis, cell cycle, angiogenesis and metastasis [8]. However, its mechanistic roles in human cancers are varied because it regulates the expression of over 400 genes that are simultaneously stimulated by multiple upstream regulators [9]. Degradation of IκBs through the proteasomal pathway after phosphorylation by IKKs activates NF-κB signaling activity. Thus, p-IKK and p-IκB can be monitored to reflect NF-κB signaling activity [10]. Previous studies have stated that blockage of NF-κB activity increases the efficiency of chemotherapy by cisplatin in ovarian cancer models [11] and suppresses ovarian cancer cell metastasis [12]. Hence, the NF-κB signaling pathway is a promising target in cancer therapy.

Transforming growth factor (TGF)-β-activating kinase 1 (TAK1) is a serine/threonine kinase and a mitogen-activated protein kinase kinase kinase that can be activated by many upstream cytokines, such as TGF-beta, IL-1beta, TNF-alpha, and toll-like receptor ligands [13]. Previous reports have shown that TAK1 may play diverse roles in various cancers by acting as tumor suppressor or oncoprotein. For instance, in liver cancer, ablation of TAK1 can cause hepatic injury, inflammation, fibrosis and carcinogenesis, which identifies TAK1 as a tumor suppressor [14]. However, in skin tumors, pancreatic cancers and colon cancers, inhibition of TAK1 up-regulates ROS, sensitizes cells to chemoresistance, and promotes apoptosis, respectively [15-17]. Indeed, the functional roles and molecular mechanisms of TAK1 in ovarian cancers remain obscure. Effects of TAK1 are mediated via phosphorylation of multiple residues in its activation loop. Many phosphorylation sites had been previously identified, such as Ser-192, Thr178, Thr184/187 and Ser412 [18-21] but only Thr184/Thr187 has been well studied in promonocytic leukemic and prostate cancer cells [22, 23]. However, which phosphorylated site required for TAK1 mediated NF-κB signaling in ovarian oncogenesis is still unclear.

In this study, we provide compelling evidence showing TAK1 is frequently overexpressed in aggressive and high-grade ovarian cancer. Importantly, TAK1 activates NF-κB signaling activity through the increased phosphorylation at Ser412, and such activated TAK1/NF-κB signaling cascade is indispensable in promoting ovarian cancer cell growth, anchorage independent growth ability, chemoresistance, as well as *in vitro* and *in vivo* metastasis in ovarian cancer. These results reveal mechanistic insights into the functional role of TAK1 in NF-κB mediated ovarian cancer aggressiveness, suggesting TAK1 is a therapeutic target for this disease.

## RESULTS

### TAK1 is frequently upregulated in ovarian cancer

**Figure 1:**
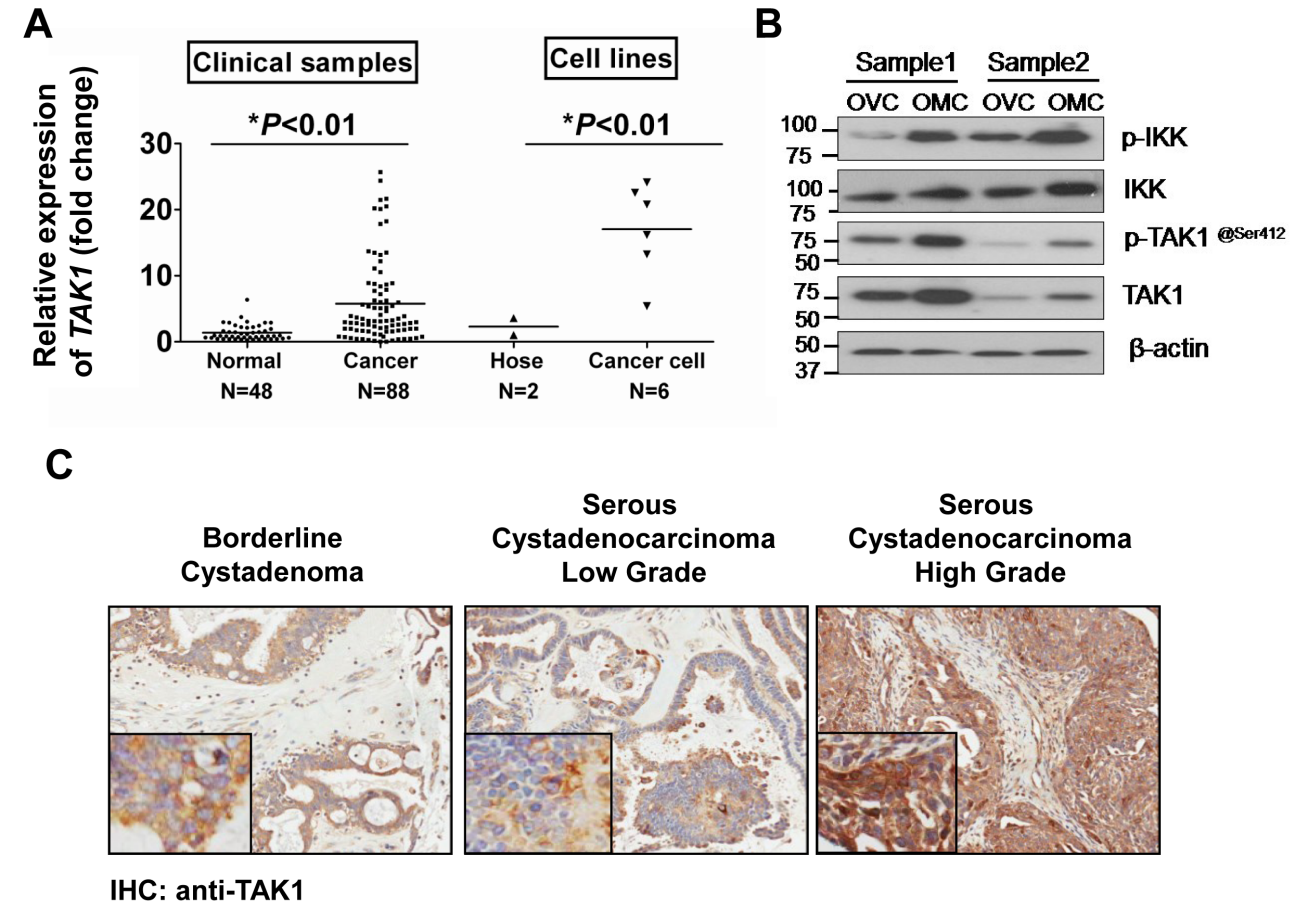
TAK1 is upregulated in high-grade and metastatic ovarian cancer (A) Q-PCR using *MAP3K* probe was performed for three times independently in normal cancer samples (n=48), ovarian cancer samples (n=88), HOSE cell lines (n=2) and ovarian cancer cell lines (n=6). The expression of *TAK1* mRNA was normalized by internal control *18S* gene. *P<0.01. (B) Representative IHC showed the staining intensity of TAK1 in borderline cystadenoma, serous low-grade and high-grade on an ovarian cancer tissue array (OVC1021) (x20). Increased immune-positive staining of TAK1 was observed along low- to high-grade ovarian cancers. (C) Western blot analysis using anti-TAK1, p-TAK1 at Ser412, IKK and p-IKK (Ser180/181) of 2 pairs of primary cultured cells from omentum (OMC) and ovary (OVC). The protein amount of loading was normalized by β-actin.

To understand the functional role and expression status of TAK1 in ovarian cancer, qPCR analysis was performed to evaluate the expression level of *TAK1* mRNA in ovarian cancer samples (n=88), normal ovaries (n=48), normal ovarian HOSE cell lines (n=2) and ovarian cancer cell lines (n=6). The results showed that TAK1 was significantly upregulated in ovarian cancer samples by 8-fold and ovarian cancer cell lines by 18-fold as compared with normal ovaries and ovarian HOSE cell lines, respectively (*P<0.01) (Figure 1A). Clinicopathological correlation indicated that overexpresson of TAK1 was remarkably associated with high-grade tumor formation (*P=0.033) (Supplementary Table 1). However, there was no significant association between TAK1 overexpression and other clinical parameters. Furthermore, Western blot and immunohistochemical analysis were conducted to evaluate the protein expression level of TAK1 in ovarian cancer cell lines and a commercial tissue array (OV1021, Pantomics) respectively. Results showed that the expression of TAK1 was obviously upregulated in ovarian cancer cell lines as compared to the HOSEs (Supplementary Figure S1). In addition, there is a progressive increase in TAK1 expression from low-grade to high-grade serous ovarian cancers (Figure 1B). By clinicopathological correlation analysis, high TAK1 expression was significantly correlated with high-grade tumor again (*P*=0. 001), in which 47.9% of high-grade tumor cases exhibited more than 6-fold overexpression of TAK1 whereas 85.4% of low-grade tumor cases showed lower expression of TAK1 (Supplementary Table 2). In addition, expression of TAK1 was highly correlated with cancer cell metastasis (*P*=0.025), in which 54% of cases demonstrated more than 6-fold overexpression of TAK1 (Supplementary Table 2). Most of serous ovarian cancers are found to have omental metastasis [24]. This implies that the cancer cells from the omentum are considered more aggressive as compared from the cancer cells still contained within the ovary. Thus, we cultured the primary ovarian cancer cells from both omentum and ovary of the same ovarian cancer patient (n = 2). Western blot analysis showed that there was a remarkable upregulation of TAK1, p-TAK1 at Ser412 and p-IKK (Ser180/181) in primary ovarian cancer cells from omentum (OMC) as compared with primary ovarian cancer cells from the ovaries (OVC) (Figure 1C). The p-TAK1 (Ser412) and p-IKK (Ser180/181) represent the activities of TAK1 and NF-κB respectively. Therefore, this result supports the findings from clinicopathological analysis, suggesting that increased TAK1 and NF-κB signaling activities are involved in aggressive ovarian cancer cells.

### TAK1 promotes ovarian cancer cell growth and anchorage independent growth ability

**Figure 2:**
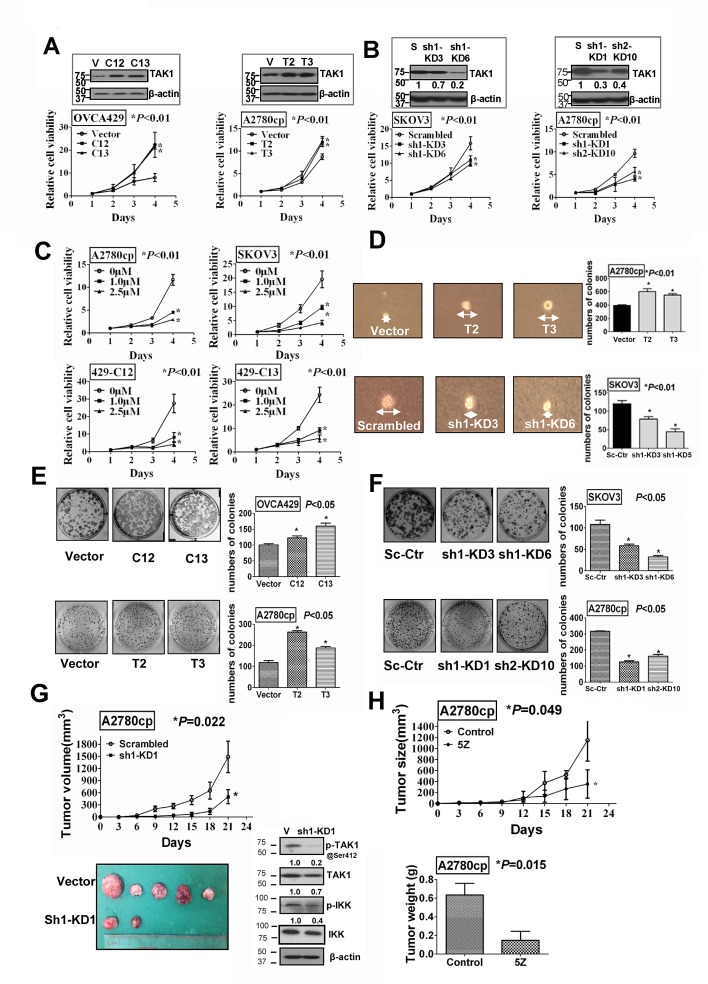
TAK1 promotes ovarian cancer cell growth *in vitro* and *in vivo* (A) Stable TAK1 over-expressing clones were established in OVCA429 and A2780cp cells. XTT cell proliferation assay demonstrated that TAK1 could remarkably increase cell proliferation in OVCA429 and A2790cp stable clones (*P<0.01). (B) TAK1 knockdown stable clones were established by shRNA in SKOV3 and A2780cp cells. Depletion of endogenous TAK1 reduced cell proliferation in SKOV3 and A2790cp stable clones (*P<0.01). (C) Treatment of TAK1 inhibitor, (5Z) -7-Oxozeaenol, (1.0 μM and 2.5 μM for 24 hours) significantly reduced cell proliferation in both ovarian cancer parental cell lines (A2780cp and SKOV3) (*P<0.01) and TAK1 stably over-expressing clones (429-C12 and 429-C13) (*P<0.01) dose dependently. (D) Soft agar assay showed that TAK1-expressing clones exhibited an increased number of colonies compared with the vector control (*P<0.01), whereas clones with depletion of TAK1 had less number of colonies as compared with the vector control (*P<0.01). Representative photos show the size of colonies under microscopy. (E) Enforced expression of TAK1 in OVCA429 and A2780cp significantly increased the number of cell colonies, (*P<0.05). (F) Depletion of TAK1 by shRNA knockdown in SKOV3 and A2780cp decreased the number of cell colonies (*P<0.05). (G) Stable TAK1 knockdown clone (Acp-sh1-KD1) showed a slower growth rate as compared with the vector control in nude mouse tumor xenograft model (*P=0. 022). Representative tumor picture was taken after sacrificed the nude mice on Day 21. Western blot analysis confirmed the expressions of TAK1, p-TAK1 at Ser412, p-IKK and IKK from the tumor tissues. (H) TAK1 inhibitor, (5Z) -7-Oxozeaenol (16mg/kg), significantly inhibited the tumor formation rate in nude mice as compared with the parental A2780cp cell control (*P=0. 049). The weight of the tumors treated with TAK1 inhibitor was approximately 3-folds lower as compared with control group (*P=0. 015).

Given that TAK1 was frequently overexpressed in ovarian cancer, in particular high-grade tumor, study of the functional role of TAK1 in ovarian cancer cells is very worthwhile. Stable TAK1-overexpressing clones were generated by transfection of TAK1 plasmids into ovarian cancer cell lines; OVCA429 (429-C12 and 429-C13) and A2780cp (Acp-T2 and Acp-T3), while stable knockdown of endogenous TAK1 was achieved in SKOV3 (SK-sh1-KD3 and SK-sh1-KD6) and A2780cp (Acp-sh1-KD1 and Acp-sh2-KD10) cells using vector-based RNAi constructs (Figure 2A-B). By XTT cell proliferation assay, enforced expression of TAK1 in OVCA429 and A2780cp showed an average 5-fold and 2-fold, respectively, higher proliferation rate than their vector controls (*P<0.01) (Figure 2A). In contrast, depletion of TAK1 in SKOV3 and A2780cp decreased the proliferation rate by ~30% and ~50%, respectively, when compared to their vector controls (*P<0.01) (Figure 2B). To further prove the importance of TAK1 in ovarian cancer cell growth, the specific TAK1 inhibitor, (5Z) -7-Oxozeaenol, was used to treat A2780cp and SKOV3 and the TAK1-over-expressing OVCA429 clones 429-C12 and 429-C13. XTT cell proliferation assay showed that inhibitor treatment suppressed the proliferation of A2780cp, SKOV3, and both 429-C12 and 429-C13, by ~66%, ~75%, and ~80%, respectively, when compared with the untreated controls (*P<0.01) (Figure 2C). In addition, soft agar assay showed that enforced expression of TAK1 increased not only the size but also the number of colonies in Acp-T2 and Acp-T3 by 1.5-fold and 1.2-fold, respectively (*P<0.01). On the other hand, depletion of TAK1 reduced both the size and number of colonies in SK-sh1-KD3 and SK-sh1-KD6 by ~40% and ~70%, respectively, (*P<0.01) as compared with their vector controls (Figure 2D). Furthermore, focus formation assay demonstrated that the TAK1-over-expressing clones in OVCA429 (429-C12 and 429-C13) and A2780cp (Acp-T2 and Acp-T3) exhibited more and larger colonies by ~1.5-fold and ~2-fold, respectively, when compared with their vector controls (*P<0.05) (Figure 2E). In contrast, the stable TAK1 knockdown clones in SKOV3 (SK-sh1-KD3 and SK-sh1-KD6) and A2780cp (Acp-sh1-KD1 and Acp-sh2-KD10) showed less and smaller colonies by ~50% and ~66%, respectively, as compared with their vector controls (*P<0.05) (Figure 2F). Taken together, these results indicate that TAK1 is capable of promoting ovarian cancer cell growth and anchorage independence.

### TAK1 increases tumor growth in vivo in ovarian cancer cells

In order to investigate the tumor forming capacity of TAK1 in ovarian cancer cells, the stable TAK1 knockdown clone (Acp-sh1-KD1) and vector control of A2780cp were selected for subcutaneous injection into nude mice at each side of flank. Acp-sh1-KD1 exhibited reduced tumor growth rate by 60% when compared to the vector control (*P=0. 022) (Figure 2G). Western blot analysis confirmed that the tumor tissues from Acp-KD1-treated mice expressed lower levels of TAK1, p-IKK and p-TAK1 at Ser412 (Figure 2G). To further confirm TAK1 in tumor growth of ovarian cancer cells, A2780cp cells expressing high levels of TAK1 were injected into nude mice subcutaneously in the presence and absence of (5Z) -7-Oxozeaenol treatment. Starting on day3, 16mg/kg (5Z) -7-Oxozeaenol or PBS (carrier solution) control, was intraperitoneally injected (i.p.) for every three days in each group of tumor bearing nude mice (n=5). After 7 times of injection, we found that there was ~70% reduction in tumor volume with (5Z) -7-Oxozeaenol treatment when compared with the PBS control on Day21 (*P=0. 049) (Figure 2H). Besides, the average tumor weight on Day21 with (5Z) -7-Oxozeaenol injection was ~75% lower as compared with the PBS control group (*P=0. 015) (Figure 2H). Altogether, these data further support the notion that overexpression of TAK1 contributes to ovarian cancer cell growth in *vivo.*

### TAK1 promotes cell migration/invasion *in vitro* and *in vivo* of ovarian cancer cells

**Figure 3:**
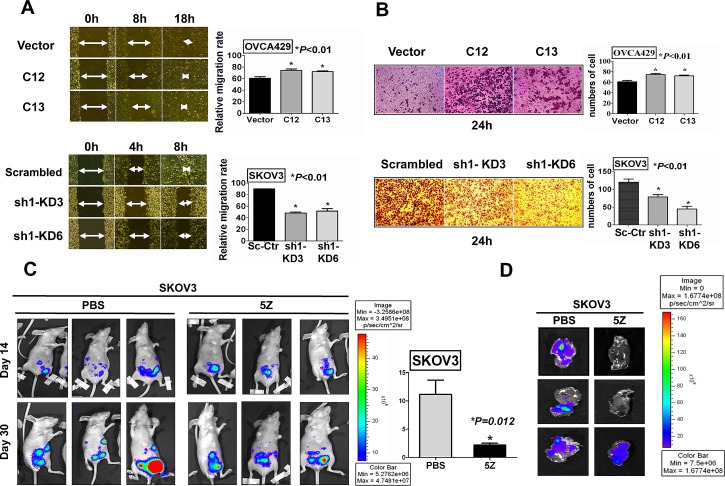
TAK1 enhances ovarian cancer cell migration/invasion *in vitro* and *in vivo* (A) Wound healing assay showed the over-expressed TAK1 exhibited a faster wound closure rate in 429-C12 and 429-C13 (*P<0.01), while knockdown of TAK1 exhibited a lower wound closure rate in SK-KD3 and SK-KD6 (*P<0.01) as compared with their vector controls. The arrows indicate the width of the wound and the relative cell migration rate is expressed as relative width of the wounds/time by bar charts. (B) Transwell cell invasion assay and the bar chart showed a higher invasive rate through Matrigel-coated membrane in TAK1 stably over-expressing clones (Acp-T2 and Acp-T3) (*P<0.01), while a lower invasive rate was observed in TAK1 knockdown clones SK-KD3 and SK-KD6 (*P<0.01) when compared to their vector controls. (C) Representative bioluminescence images on Day14 and Day30 of nude mice in PBS (carrier solution) group and TAK1 inhibitor ((5Z) -7-Oxozeaenol, 16mg/kg, 5 injections) group; PBS (carrier solution) group (n=3) presented significant higher relative bioluminescence index on Day30 as compared to the TAK1 inhibitor group (n=3), *P*=0.012; (D) Representative biofluorosence imaging of the livers. On Day30 for SKOV3 control group with PBS (carrier solution) and TAK1 inhibitor treatment group.

High cell proliferation, migration and invasion are salient features of aggressive high-grade ovarian tumors[25]. On the other hand, previous studies have shown that TAK1 is required for bone metastasis [26] and inhibition of TAK1 blocks cancer cell invasion and metastasis in breast cancer[27]. Hence, we postulated that TAK1 overexpression is able to promoting cell migration and invasion of ovarian cancer cells. Wound healing assay was firstly performed to examine the function of TAK1 in the cell migration capacity of ovarian cancer cells. Upon treatment of Mitomycin C to exclude the factor of increased cell growth, we observed a faster wound closure rate in 429-C12 and 429-C13 by 1.4-fold and 1.3 fold, respectively, when compared to their vector controls (*P<0.01). Conversely, knockdown of endogenous TAK1 in SK-sh1-KD3 and SK-sh1-KD6 significantly reduced the cell migration rate by 50% and 40%, respectively, as compared with their vector controls (*P<0.01) (Figure 3A). Furthermore, using Transwell invasion assays, we demonstrated that there was a remarkable increase by 1.5-fold and 1.3-fold in cell invasion rate in Acp-T2 and Acp-T3, respectively, as compared with vector controls (*P<0.01). In contrast, the numbers of cells invading through matrigel in Transwell invasion assays were significantly reduced in SK-sh1-KD3 and SK-sh1-KD6 by 30% and 60%, respectively, as compared with vector controls (*P<0.01) (Figure 3B). To further confirm these functional roles of TAK1, a mouse model for *in vivo* study of ovarian cancer metastasis was conducted. The GFP-luminescence labelled SKOV3 cells (CMV-GFP-T2A-Luciferase) were injected (intraperitoneally) i.p. into 6 nude mice. After 14 days, bioluminescence images were taken to record the start point (Figure 3C). Then the mice were separated into two groups; one group received intraperitoneal injections of TAK1 inhibitor, (5Z) -7-Oxozeaenol (16mg/kg), while the control group was injected with PBS only. After 5 injections, the bioluminescence imaging of the PBS group displayed prominent tumor size growth with an average 10-fold increase, whereas only 3.2-fold increase could be observed in the TAK1 inhibitor treated group on day 30 (*P=0.012) (Figure 3C). In addition, the livers of the mice were collected at the end of the experiment and examined with biofluorosence. Results showed that the livers in PBS group demonstrated stronger intensity of GFP signal as compared to the TAK1 inhibitor group, indicating that the TAK1 inhibitor could inhibit SKOV3 cells metastasized to livers of mice (Figure 3D). Taken together, these data suggest that inhibition of TAK1 activity is able to impair *in vitro* and *in vivo* ovarian cancer cell motility and metastasis.

### TAK1 sensitizes ovarian cancer cells to cisplatin-induced cytotoxicity

**Figure 4:**
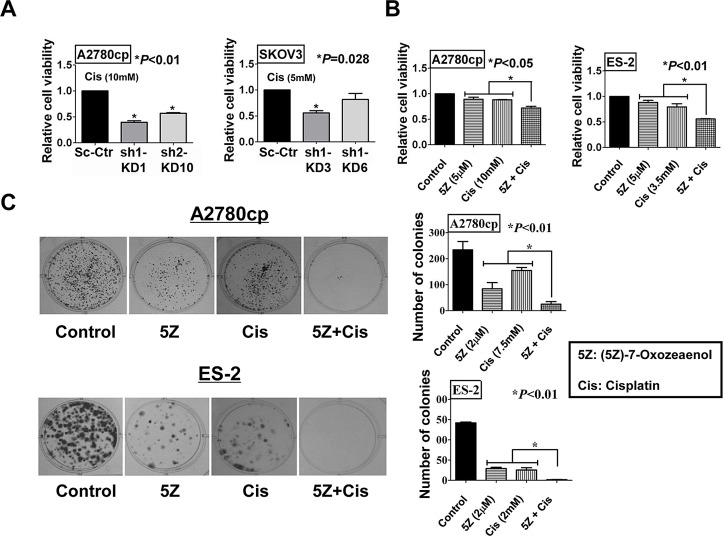
Inhibition of TAK1 sensitizes ovarian cancer cells to cisplatin-induced cell apoptosis (A) XTT proliferation assay was performed to detect the effect of cisplatin on cell proliferation with ovarian cancer cells. The bar chart shows that TAK1 knockdown stable clones were more sensitive to cisplatin treatment as compared to vector control. Stable clones Acp-KD1 and Acp-KD10 were treated with 10mM cisplatin for 48h (*P<0.01), and SK-KD3 and SK-KD6 were treated with 5mM cisplatin for 48h (C3, *P=0. 028). (B) The bar chart showed that TAK1 inhibitor, (5Z)-7-Oxozeaenol (5μM), could sensitize ovarian cancer A2780cp (*P<0.05) (cisplatin: 10mM) and ES-2 (*P<0.01) (cisplatin: 3.5mM) to cisplatin-induced cell apoptosis. The results were obtained from XTT cell proliferation assay in a 48-hour time course. (C) Focus formation assay was performed and showed that TAK1 inhibitor, (5Z) -7-Oxozeaenol (2μM), could sensitize ovarian cancer A2780cp (*P<0.01) (cisplatin: 7.5mM) and ES-2 (*P<0.01) (cisplatin: 2mM) to cisplatin-induced cell apoptosis as compared with their controls or each single treatment. Representative photos were taken to show the number and size of the colonies.

Previous studies have reported that TAK1 increases chemoresistance via the NF-κB pathway and targeting TAK1 has been shown to be a potential therapeutic approach to reducing the chemoresistance of pancreatic cancer [16, 28]. However, the functional role of TAK1 in ovarian cancer chemoresistance is unknown. To define whether inhibition of TAK1 can sensitize ovarian cancer cells to cisplatin-induced cell apoptosis, XTT cell proliferation assay was performed in the TAK1 knockdown clones SK-sh1-KD3, SK-sh1-KD6, Acp-sh1-KD1 and Acp-sh2-KD10. Notably, treatment of TAK1-depleted A2780cp clones (Acp-sh1-KD1 and Acp-sh2-KD10) with cisplatin (MERK MILLIPORE, Billerica, MA; dissolved in DMSO), and TAK1-depleted SKOV3 clones (SK-sh1-KD3 and SK-sh1-KD6) with cisplatin led to a remarkable decrease in cell viability by 60% and 40% in Acp-sh1-KD1 and Acp-sh2-KD10, respectively (**P<0.01), and 40% in SK-sh1-KD3 (*P=0.028) as compared to their vector controls (Figure 4A). In addition, (5Z)-7-Oxozeaenol co-treatment with cisplatin decreased the cell viability of A2780cp and ES-2, which express high levels of TAK1 by 25% and 50%, respectively (*P<0.01) (Figure 4B). Moreover, focus formation assay confirmed that co-treatment of (5Z) -7-Oxozeaenol could cause less number and smaller size of colonies in both cell lines treated with cisplatin (*P<0.01) (Figure 4C). These findings confirm that the upregulation of TAK1 is involved in cisplatin-resistance of ovarian cancer cells.

### Ser412 phosphorylation is required for TAK1 to activate NF-κB signaling in ovarian cancer

**Figure 5:**
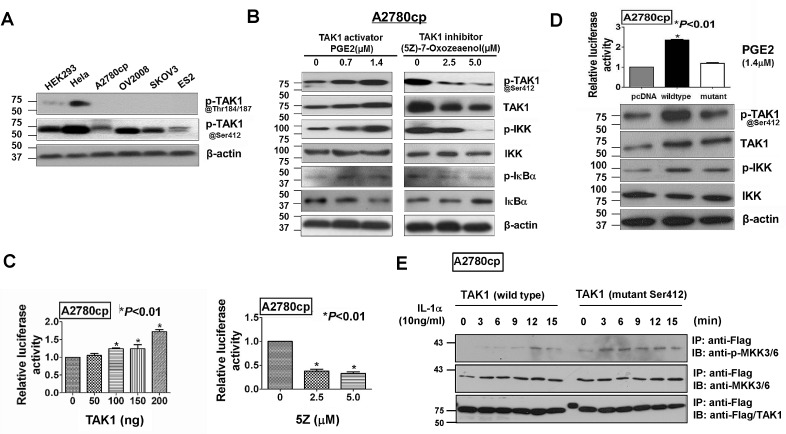
TAK1 exerts its functional effects through activation of NF -κB pathways by phosphorylation at Ser412 (A) Western blot analysis demonstrated that p-TAK1 at Thr184/187 was just found in HEK293 and Hela cells only, while p-TAK1 at Ser412 was generally found in all cell types including ovarian cancer cell lines (n=4). (B) Western blot analysis showed the phosphorylation of p-TAK1 (Ser412) was increased concomitantly with p-IKK (Ser180/181) and p-IκB (Ser32/36) as upon treatment of TAK1 activator, PGE2, in ovarian cancer cells, whereas treatment with TAK1 inhibitor, (5Z) -7-Oxozeaenol, completely diminished the phosphorylation of p-TAK1 (Ser412), p-IKK (Ser180/181) and p-IκB (Ser32/36) concentration dependently. The level of IκBα was reduced by PGE2 and increased upon treatment of (5Z) -7-Oxozeaenol. Cells were treated by PGE2 or (5Z)-7-Oxozeaenol for 24 hours before harvested for Western blotting. The protein amount of loading was normalized by β-actin. (C) NF-κB luciferase reporter assay showed that TAK1 increased the luciferase activity of NF-κB in A2780cp concentration dependently (**P<0.01). Co-treatment of TAK1 inhibitor, (5Z) -7-Oxozeaenol (2.5 μM and 5.0 μM for 24 hours), decreased the luciferase activity of NF-κB in TAK1 transfected A2780cp cells (*P<0.01). (D) Upon treatment of PGE2 (1.4 μM), the point mutations at Ser412 of TAK1 had the similar expression level of TAK1 protein as wild type TAK1, however, the phosphorylation of p-TAK1 (Ser412) was totally abrogated. The protein amount of loading was normalized by β-actin. The above experiments were performed thrice independently. (E) TAK1 *in vitro* kinase assay. The pcDNA/Flag-TAK1 and pcDNA/Flag-mutTAK1 were transfected into A2780cp cells respectively. After 48 hours, Human IL-1α (10ng/ml) was used to treat the transfected cells with various time points. Both wild-type and mutant TAK1 were immunopreciptated (IP) from cell lysates, and TAK1 kinase activity was examined by evaluating the level of Phospho-MKK6 using immunoblotting (IB). The input of total MKK6 and Flag/TAK1 or Flag/mutant TAK1 were checked by immunoblotting using anti-MKK6 and anti-Flag respectively.

Since TAK1 was confirmed to be upregulated in ovarian cancer and its level well correlated with tumor progression, the molecular mechanism of TAK1 action deserves further investigation. The downstream effects of TAK1 are mediated via phosphorylation of multiple residues in its activation loop [18]. Our western blot analysis revealed that thephosphorylation of p-TAK1 (Thr184/187) was very low, whereas the phosphorylation of p-TAK1 (Ser412) was dominately expressed in all the ovarian cancer cell lines (Figure 5A). It has previously been reported that the NF-κB pathway is implicated in the regulation of cell migration and invasion [11, 12]. Since TAK1 takes many functional roles in ovarian cancer progression such as cell proliferation, migration/invasion and importantly, Kobayashi *et al*. has reported that the phosphorylation of the Ser412 residue in TAK1 could activate NF-κB through induction of IκBα degradation [21]. Therefore, we hypothesized that NF-κB signaling might also be activated by TAK1 via PKA-pathway mediated IκBα degradation in ovarian cancer cells. As expected, our western blot results showed that PGE2 could increase the phosphorylation of Ser412 in TAK1 that in turn, elevated the phosphorylation of p-IKK (Ser176/180) and p-IκB (Ser32/36), and reduced the level of IκBα in A2780cp cells (Figure 5B). Conversely, treatment of A2780cp cells with the TAK1 inhibitor (5Z) -7-Oxozeaenol showed an opposite results to the use of PGE2 (Figure 5B). In addition, using NF-κB luciferase reporter assay, NF-κB signaling activity was shown to increase from 1.2-fold to 2-fold when A2780cp cells were transiently transfected with the TAK1-expressing plasmid pCMV-HA-TAK from 0 ng to 200 ng (*P<0.01) (Figure 5C). In contrast, NF-κB signaling activity in A2780cp cells was reduced by 60% to 65% upon treatment with (5Z) -7-Oxozeaenol from 0 M to 5.0 μM (*P<0.01) (Figure 5C).

To better understand whether phosphorylation at Ser412 is critically required for TAK1 function, a mutant TAK1 plasmid (pCDH-TAK1-mut) was generated by PCR-based site-directed mutagenesis the Ser412→Ala mutant TAK1 (Supplementary Figure S2) [21]. Western blot analysis confirmed that ectopic expression of the pCDH-TAK1-mut plasmid in A2780cp cells could not increase phosphorylation at Ser412 of TAK1 but had two-fold less of the phosphorylation of p-IKK (Ser176/180) than that of using the wild-type TAK1 plasmid upon treatment of PGE2 (1.4μM) (Figure 5D).

**Figure 6:**
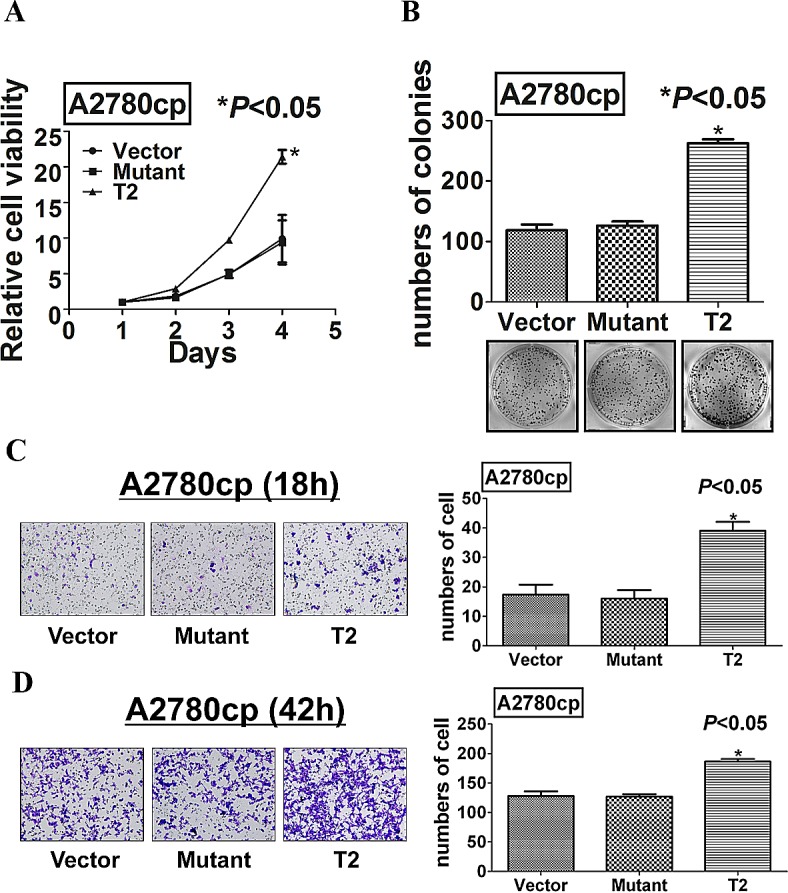
Mutation at Ser412 of TAK1 completely abrogates its function in ovarian cancer aggressiveness (A) XTT cell proliferation assay, (B) focus formation, (C) Transwell migration, and (D) Transwell invasion assays demonstrated that the point mutation at Ser412 completely impaired the functional effects of TAK1 in promoting cell growth, cell migration and invasion as compared with wild-type TAK1 overexpressing A2780cp cells (T2) (*P<0.05).

Accumulating evidence has suggested that TAK1 forms a complex with TAB1 and TAB2/3 at N- and C-termini respectively (Supplementary Figure S2) [29, 30], we questioned whether the phosphorylation at Ser412 residue in TAK1 could modulate the kinase activity of TAK1. Hence, we performed *in vitro* kinase assay for TAK1 activity according to Yang *et al* [28]. Upon induction of IL-1α in A2780cp transfected with the wild-type TAK1 palsmid (pcDNA/Flag-TAK1) and the mutant TAK1 plasmid (pcDNA/Flag-mutTAK1), the immunoprecipitated wild-type TAK1 could remarkably phosphorylate its downstream target, MKK6, while the immunoprecipitated mutant TAK1 had relatively lower capacity in phosphorylation of MKK6 (Figure 5E). This infers that the phosphorylation of TAK1 at Ser412 increase the kinase activity of TAK1 in ovarian cancer cells. Furthermore, pCDH-TAK1-Mut, when stably transfected into A2780cp, could not exert any increased higher capacities in cell proliferation, migration and invasion when compared with vector controls or as same as wild-type TAK1 overexpressing cells (Figure 6A-D). These data give the first report that phosphorylation of TAK1 at Ser412, instead of Thr184/187, is critically required for activation of the NF-κB pathway and its oncogenic properties.

## DISCUSSION

Recent studies have revealed that the *personalized* gene-targeted. cancer therapy shows promising results in improving the survival and quality of life of patients suffering advanced-stage and high-grade tumors [4-7]. Therefore, a better understanding of molecular mechanisms underlying ovarian cancer oncogenesis is urgently needed for developing this therapeutic approach. In this study, we have provided overwhelming evidence that TAK1 plays an oncogenic role in promoting cell proliferation, anchorage-independent growth ability, cell migration/invasion, and chemoresistance, as well as tumor growth/metastasis of ovarian cancer cells in both *in vitro* and *in vivo* models. Our findings indicate that TAK1 was significantly overexpressed in ovarian cancers, in particular high-grade and metastatic tumors. More importantly, we identify phosphorylation at Ser412 is critically required for TAK1 in activating of NF-κB activity and its tumorigenic capacities in ovarian cancer cells.

Previous studies have reported that the increased NF-κB activity is involved in metastatic serous ovarian carcinoma and targeting NF-κB pathway is a promising therapeutic approach to high-grade metastatic ovarian cancer [31, 32]. This indicates that NF-κB signaling acts as a master regulator in aggressive ovarian cancers. However, there is a lack of studies showing the upstream regulation of NF-κB signaling in carcinogenesis. In this study, we have revealed that TAK1 is frequently found in high-grade tumors with metastatic tendency, suggesting that TAK1 shares similar functional properties as the NF-κB pathway in aggressive human cancers. On the other hand, previous studies have documented that TAK1 can upregulate the NF-κB signaling which in turn increases the chemoresistance of human cancers [28, 33, 34]. Hence, we postulate that TAK1 promotes ovarian cancer cell growth, cell migration and invasion, as well as cisplatin-resistance via modulation of NF-κB signaling. Indeed, our results using Western blotting and NF-κB promoter luciferase assays clearly show that TAK1 is able to modulate NF-κB signaling activity, whereas either co-treatment with TAK1 inhibitor, (5Z) -7-Oxozeaenol, or shRNA-mediated TAK1 knockdown abrogates TAK1's effect on NF-κB signaling activity in ovarian cancer cells.

TAK1 is a mitogen-activated protein kinase kinase kinase that can be activated by various upstream cytokines through formation of a protein-signaling complex which consists of a variety of proteins such as TAB1, TAB2/3 and TRAFs [35]. Thus, TAK1 can exert distinct functions in different cell types depending on the nature of the upstream inducing signals [35] This makes TAK1 a double-edged sword in different types of human cancers [36]. For instance, suppression of TAK1 is required to promote prostate cancer tumorgenesis [37], while activation of TAK1 leads to hepatocyte apoptosis in liver cancer through activation of JNK signaling and NF-κB-independent function of NEMO, indicating that TAK1 is a tumor suppressor in some subtypes of human cancers [14, 37, 38]. On the other hand, TAK1 has been shown to enhance oncogenic capacities in liver cancer, lung cancer, pancreatic cancer and head and neck cancer via activation of the NF-κB pathway [26, 33, 39-41]. These evidences suggest that TAK1 plays dual roles in oncogenesis; tumor suppressor and oncogene, depending on the downstream signaling pathway that is activated and therefore dominating in a particular cancer cell type. In this study, we have demonstrated that NF-κB signaling is indispensable for TAK1 function in ovarian cancer cells. It is well known that the phosphorylation of TAK1 can subsequently activate IKK leading to the activation of NF-κB [14] and in cell metastasis of breast and renal cell cancers [26, 42]. In fact, using the TAK1 inhibitor (5Z) -7-Oxozeaenol, as well as gain- and loss-of-function genetic modifications of TAK1, we have provided strong evidences to support that TAK1 is required for NF-κB activation in ovarian cancer. Hence, these findings point to TAK1 being an oncogenic gatekeeper for NF-κB signaling, which is a key signaling pathway in governing ovarian cancer tumorigenesis.

It has been demonstrated that TAK1 kinase activity is regulated by multiple post-translational modifications of TAK1 and TABs [43]. Many phosphorylation sites had been identified previously, such as Ser192, Thr178, Thr184/187 and Ser412 [18-21]. Ser192 autophosphorylaion was first identified to be involved in IL-1 induced activation of NF-κB through the interaction of TAB1 at N-terminus of TAK1 kinase domain [18]. Dual phosphorlation of Thr178 and Thr184 was required for IL-1- and IL-6-mediated activation of both the NF-κB and JNK pathways [19]. In this study, we have surprisingly found that Thr184/187 of TAK1 is not the core phosphorylated sites in ovarian cancer cells. Instead, we identify the phosphorylation site at the Ser412 residue as being critically required for TAK1 in mediating NF-κB activity in ovarian cancer cells. Although the Ser412 residue is not in the kinase domain of TAK1, it has been shown to be interacted with TAB2 at the C-terminus of TAK1 and could be phosphorylated by PKA pathway [21]. cAMP/PKA signaling is an oncogenic signaling and is involved in ovarian cancer progression [44] [45]. Importantly, cAMP/PKA signaling has been reported to promote NF-κB activity[46, 47]. Indeed, in consistent with the previous finding of Kabayashi *et al*. [21], our data showed that the increased phosphorylation of Ser412 by treatment of PGE2 led to the increased degradation of IκBα (elevated p-IκB (Ser32/36)) and increased phoshphorylation of p-IKK (Ser176/180) (NF-κB activity). This has been further confirmed using NF-kB luciferase reporter assay. In contrast, mutation by Ser412→Ala aboragated the capacity of TAK1 in promoting NF-κB activity as well as its associated oncogenic properties in ovarian cancer cells. On the other hand, it has been shown that the phosphorylation of TAK1 Ser412 by PKA may influence the signaling complex formation (TAK1-TAB1-TAB2/TAB3) induced by the various ligands including IL-1α in Ser192 of TAK1 [18, 21]. To examine whether the phosphorylation of Ser412 affects the complex confirmation as well as the kinase activity of TAK1, an *in vitro* TAK1 kinase was performed to examine the capacity of the wild-type or the Ser412 mutant TAK1 in modulating TAK1 activity under IL-1α induction. As expected, we found that Ser412 phosphorylation has not only activated NF-κB through degradation of IκBα but also alters IL-1α-induced TAK1 activity. However, we still don't know how the phosphorylation of Ser412 affects the signaling complex formation (TAK1-TAB1-TAB2/TAB3) and the kinase activity. Hence, further study in examining the underlying mechanism is warranted. However, to the best of our knowledge, this is the first report showing that the phosphorylation of Ser412 residue in TAK1 is essential for the activation of NF-κB signaling in ovarian cancer cells.

In conclusion, our findings suggest that the upregulation of TAK1 and the phosphorylation at Ser412 residue in TAK1 is required for enhancing NF-κB-dependent oncogenic capacities in aggressive high-grade ovarian cancer. Therefore, targeting TAK1 may be a potential therapeutic approach in the personalized molecular therapy in ovarian cancer.

## MATERIALS AND METHODS

### Ovarian cancer samples and cell lines

A total of 88 tumor samples surgically resected from primary ovarian cancer patients and 48 normal ovary samples from benign diseases were randomly selected for this study. The histologic subtypes and disease stages of the tumors were classified according to International Federation of Gynecology and Obstetrics criteria. The use of clinical specimens was approved by the local institutional ethics committee (IRB no. UW11-298). Four ovarian cancer cell lines (high-grade serous subtype); A2780cp, (kindly provided from Professor Benjamin Tsang, The University of Ottawa), SKOV3 and OVCA429 (ATCC, Rockville, MD, USA) [48], one ovarian cancer cell line (clear cell subtype), ES-2, (ATCC), as well as HEK293 cells were cultured in Dulbecco's modified Eagle medium (DMEM) (Gibco-BRL, Gaithersburg, MD) with 10% Fetal Bovine Serum (FBS) (Gibco-BRL, Gaithersburg, MD), penicillin and streptomycin (100 units/ml) at 37^0^C in incubator with 5% CO_2_. The cell line authentication was done by in-house STR DNA profiling analysis. Two pairs of primary cultured cells from the same patients from both omentum (OMC) and ovaries (OVC) were obtained in Queen Mary Hospital under human ethics (HKU/HA HKW IRB number: UW 11-298). The primary culture cells were incubated with a mix medium of MCD (Invitrogen) and 199 (Invitrogen) with a proportion of 1:1. All the experiments were performed after 10 days incubation.

### Plasmids and Cell Transfection

Stable TAK1-overexpressing clones were generated by either transfection of pCMV-HA-TAK (kindly provided by Kuni Matsymoto Lab, Acp-T2 and Acp-T3) or pCDH-Tag-1-TAK1 lentiviral plasmid (429-C12 and 429-C13) constructed by PCR amplification of the TAK1 ORF from pCMV-HA-TAK followed by subcloning into the pCDH-CMV-MCS-EF1-copGFP (SBI). TAK1 shRNAi constructs (HuSHTM Company, Japan) were used to knockdown endogenous TAK1 in TAK1-overexpressing ovarian cancer cell lines. Stable mutant Ser412 TAK1 clones were generated by transfection of A2780cp cells with the pCDH-TAK1-mut plasmid, which was constructed by PCR-based site-directed mutagenesis of Ser 412 to Ala 412 using designed primers according to Kobayashi *et al*. [21]. GFP-luminescence labeled stable clones were generated by lenti-virus transfection of Lenti-reporter plasmid, CMV-GFP-T2A-Luciferase (BLIV101PA-1) (System Biosciences, Mountain view, CA).

### Quantitative Reverse Transcription-Polymerase Chain Reaction (qPCR)

qPCR was performed using the oligonucleotides/TagMan probes provided for TagMan Gene Expression Assays (Applied Biosystems, Foster City, CA) (*TAK1*, Assay ID: HS01105682_m1; *18S*, Catalog Number: 4310893E) in an ABI 7500 system according to the manufacture's instruction. Expression levels of target genes were quantified against endogenous *18S level* using the comparative C_T_ method. The 7500 System SDS Software was used for data analysis.

### Western blot and Immunohistochemical (IHC) analyses

For Western blot analysis, cells were lysed with cell lysis buffer (Cell Signaling Technology, Danvers, MA) containing protease inhibitor cocktail (Roche, Indianapolis, IN) and phenylmethylsulfonyl fluoride (Sigma, St. Louis, MO). Samples were resolved by SDS-PAGE and electroblotted onto Immobilon-P Transfer Membrane (Merk Millipore, Billerica, MA). Blots were blocked with 5% skim milk, followed by incubation with antibodies against TAK1, Phospho-TAK1 (Ser412), Phospho-TAK1 (Thr184/187), Phospho-IKKα/β (Ser180/181), IKK, Phospho- IκBα (Ser32/36), IκBα, Phospho-MKK3 (Ser189)/MKK6 (Ser207) (Cell Signaling Technology, Danvers, MA); MKK6 (R&D Systems, Minneapolis, MN); GFP (Santa Cruz Biotechnology, Santa Cruz, CA) or β-actin (Sigma, St. Louis, MO). Blots were then incubated with goat anti-rabbit or anti-mouse secondary antibodies that are conjugated with horseradish peroxidase (Amersham Pharmacia Biotech, Piscataway, NJ) and signals visualized by enhanced chemiluminescence.

Immunohistochemical staining for TAK1 was performed on an ovarian cancer tissue array (OVC1021; Pantomics, Inc.). The section was immunostained with primary polyclonal anti-TAK1 antibody (Cell signaling Technology, Danvers, MA, USA) in 1:50 dilution. For negative controls, the primary antibody was replaced with TBS. The immunoreactivity score for each case was calculated as previously protocol [49].

### Luciferase reporter assay

Luciferase Reporter Assay was performed using the DLR Assay kit (Promega, Madison, WI) according to the manufacturer's instructions. Cells were cultured on 6-well plates and were transfected with plasmids two days before the luciferase assay.

### *In vitro* Kinase assay

To examine the TAK1 activity, an *in vitro* kinase assay was modified from Yang *et al.* [28]. The wild type TAK1 and mutant TAK1 from pCMV-HA-TAK and pCDH-TAK1-mut plasmids were subcloned into pcDNA3.1(+)/3xFlag vector to generate pcDNA/Flag-TAK1 and pcDNA/Flag-mutTAK1. Both plasmids were transfected into A2780cp cells, respectively an cell lysates were harvested after 48 hours and treated with Human IL-1α (Peprotech, Rocky Hill, NJ). Cell lysates were prepared by Cell Lysis Buffer (Cell Signaling) and immunoprecipitated with anti-Flag (Sigma) followed by overnight incubation with protein A/G-conjugated beads (Santa Cruz) at 4^0^C. After 3 times of washing using Cell Lysis Buffer, an *in vitro* kinase reaction was performed at 30°C for 90 min in 20 μl Kinase Buffer (Cell Signaling) containing ATP (200μM) and 1 μg of unactive MKK6/SKK3 protein (Millipore, Billerica, MA). The activities of wild type TAK1 and mutant TAK1 were determined by the levels of phospho-MKK6 using Western blotting.

### *In vitro* functional assays

Cell proliferation kit (XTT) (Roche, Indianapolis, IN, USA) was used to measure cell viability according to the manufacturer's manual [50]. Focus formation assay was performed as a more long-term proliferation assay and soft agar assay was performed to detect cell anchorage-independent growth ability. Wound healing assay and a Cell Migration Assay kit (MILLIPORE, Cat. No. ECM508) were used to detect cell migration ability. Quantification of cell invasion was performed using QCM™ 24-Well Colorimetric Cell Invasion Assay Kit (Chemicon International, Temecula, CA, USA). All experiments were done in triplicates and results were presented as the mean ± SD.

### Tumor xenograft mouse model

A2780cp-sh1-KD1 and its scrambled control cells at a concentration of 1×10^6^ cells/100μl were injected subcutaneously into 5-week female BALB/c nude mice. For Acp-sh1-KD1 and vector control injection, tumor formation in nude mice (n=5) was monitored for every 3 days since Day 3. For the A2780cp cell injection group, the TAK1 inhibitor, (5Z) -7-Oxozeaenol (16mg/kg) (Sigma) (in carrier solution which consists of 10% DMSO in PBS), was injected into the mice in peritoneal every three days., was administered by intraperitoneal (i.p.) injections for every two days with total of 7 injections into five mice from Day 3. As a control group, the carrier solution only was administrated i.p. for the same time of treatment. Tumor sizes were measured using slide calipers and were calculated by the following formula: volume = (width) ^2^ × length × π/6. Tumor growth curves were plotted from the mean volume±SEM of tumors from 5 mice. Side effects such as body weight changes were monitored closely. For *in vivo* mouse model of metastasis, the pure GFP-luminescence tagged SKOV3 cells (1×10^6^ cells/100 μl) were mixed with 100 μl Matrigel Matrix (BD Biosciences, San Jose, CA) and intraperitoneally injected into 6 nude mice. TAK1 inhibitor, (5Z) -7-Oxozeaenol (16mg/kg) (in carrier solution), was administered i.p. for every three days with total 5 injections into 3 mice. For the control group, the carrier solution was injected i.p. only. The ovarian cancer cells (with GFP-labelled) metastasized to livers of mice were analyzed by biofluorscence imaging. Both bioluminescence and biofluorescence imagings were performed using the Xenogen IVIS 100 system and analyzed using Living Imaging^(R)^, version 2.50.1. All the animal experiments were approved by the University of Hong Kong Committee on the Use of Live Animals in Teaching and Research (CULATR No. 2053-09).

### Statistical analysis

Receiver operating characteristic (ROC) curve was used to determine cut-off points for qPCR and IHC results. The clinicopathological analysis between the expression of TAK1 and clinical parameters was analyzed by Crosstabs and Pearson Chi-Square test. The Student's *t*-test was used to analyze cell viability, migration/invasion and *in vivo* tumor growth results. Statistical analyses were performed using the SPSS 13.0 software (SPSS). *P*-values of less than 0.05 were considered significant in all tests. All data were expressed as mean ± SD.

### SUPPLEMENTAL MATERIAL FIGURES AND TABLES


