# Supplementary material for: Elevated TAK1 augments tumor growth and metastatic capacities of ovarian cancer cells through activation of NF-κB signaling
Source: Oncotarget. 2014 Jul 27;5(17):7549–62. doi: 10.18632/oncotarget.2273 (PMC4202143; doi:10.18632/oncotarget.2273)
Supplement: Supplementary file 1 [file oncotarget-05-7549-s001.pdf]

Elevated TAK1 augments tumor growth and metastatic capacities of ovarian cancer cells through activation of NF-κB signaling

Supplementary Material

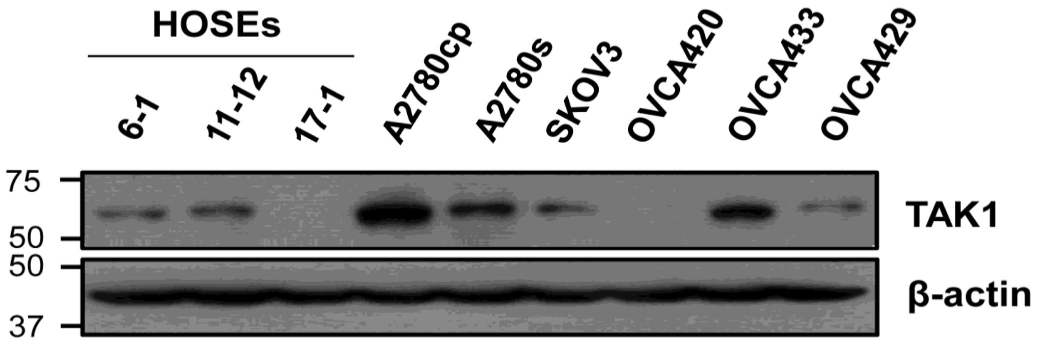

Supplementary Figure S1: Western blot analysis showed the expression of TAK1 (78kDa) in a panel of ovarian cancer cell lines (n=6) and HOSEs (n=3).

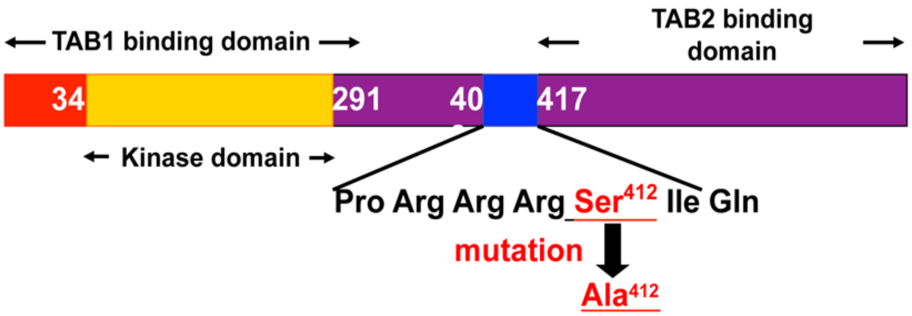

Supplementary Figure S2: The plasmid pCDH-TAK1-mut was constructed by changing Ser412 to Ala412.

Supplementary Table 1: Clinicopathological correlation of the mRNA expression level of *TAK1* evaluated by qPCR analysis in ovarian cancer samples. The 3-fold TAK1 expression was set to be cut-off point according to ROC curve analysis.

		**TAK1 expression**		
**Characteristics**	**Total**	**≤ 3 folds**	**>3 folds**	***P***
**All cases**	70	33	37	
**Age**				
**<=50**	38	18 (47.3)	20 (52.6)	
**>50**	32	15 (46.9)	17 (53.1)	0.967
**Stage**				
**Early**	24	12 (50.0)	12 (50.0)	
**Late**	45	21 (46.7)	24 (53.3)	0.792
**Grade**				
**Low**	32	18 (56.3)	14 (43.8)	
**High**	25	7 (28.0)	18 (72.0)	**0.033***
**Histology**				
**Clear cell**	17	9 (52.9)	8 (47.1)	
**Others**	53	24 (45.3)	29 (54.7)	0.967
**Recurrence**				
**+**	36	18 (50.0)	18 (50.0)	
**-**	29	11 (37.9)	18 (62.1)	0.554

Supplementary Table 2: Clinicopathological correlation of TAK1 level analyzed by IHC on an ovarian cancer tissue array (OVC1021, Pantomics. Inc.). The 6-fold TAK1 expression was set to be cut-off point according to ROC curve analysis.

TAK1 expression [fold change]				
**Characteristics**	**Total**	**≤ 6 folds**	**>6 folds**	**p**
**All cases**	97	67 (69.1)	30 (30.9)	
**Stage**				
**Early**	73	54 (74.0)	19 (26.0)	
**Late**	24	13 (54.2)	11 (52.4)	0.080
**Grade**				
**Low**	48	41 (85.4)	7 (14.6)	
**High**	48	25 (52.1)	23 (47.9)	**0.01** *
**Metastasis**				
**No**	73	55 (75.3)	18 (24.7)	
**Yes**	24	11 (46.0)	13 (54.0)	**0.025***
